# Synergistic effect of graphene and silicon dioxide hybrids through hydrogen bonding self-assembly in elastomer composites†

**DOI:** 10.1039/c8ra01659c

**Published:** 2018-05-16

**Authors:** Shuai Zhao, Shicheng Xie, Peipei Sun, Zheng Zhao, Lin Li, Xiaoming Shao, Xiaolin Liu, Zhenxiang Xin

**Affiliations:** Key Laboratory of Rubber-Plastics, Ministry of Education, Shandong Provincial Key Laboratory of Rubber-Plastics, School of Polymer Science and Engineering, Qingdao University of Science and Technology Qingdao 266042 China lyzhsh@163.com qustlilin@163.com

## Abstract

A novel graphene–silicon dioxide hybrid (HGS) was prepared by plant polyphenol-tannic acid (TA) functionalized pristine graphene (G-TA) and primary amine-containing silane coupling agent modified SiO_2_ (Si–NH_2_). Through strong hydrogen-bonding interaction between the phenolic hydroxyl groups on G-TA and primary amine groups on Si–NH_2_, SiO_2_ was uniformly loaded to the surface of graphene. Due to the synergistic dispersion effect of graphene and SiO_2_, which prevents restacking and re-aggregating of both graphene and SiO_2_, HGS hybrids were distributed evenly in the natural rubber (NR) matrix (HGS@NR). Simultaneously, the surface roughness of graphene after loading SiO_2_ and the interfacial interaction between the HGS hybrid and NR matrix were substantially improved. Due to the good dispersion and strong interface, the overall properties of HGS@NR nanocomposites are drastically enhanced compared with those of GS@NR nanocomposites prepared by dispersing the blend of unmodified graphene and SiO_2_ (GS) in NR. The HGS@NR nanocomposites possess the highest tensile strength up to 27.8 MPa at 0.5 wt% and tear strength of 60.2 MPa at 0.5 wt%. Thermal conductivities of the HGS@NR nanocomposites were found to be 1.5-fold better than that of the GS@NR nanocomposites. Also, the HGS@NR nanocomposites exhibit excellent abrasive resistant capacity that is nearly 2-fold better than that of the GS@NR nanocomposites. These results suggest that HGS has great potential in high-performance nanocomposites and a new strategy of constructing the efficient graphene–SiO_2_ hybrid fillers has been established.

## Abstract 2

A novel graphene–silicon dioxide hybrid (HGS) was prepared by plant polyphenol-tannic acid (TA) functionalized pristine graphene (G-TA) and primary amine-containing silane coupling agent modified SiO_2_ (Si–NH_2_).

## Graphical abstract

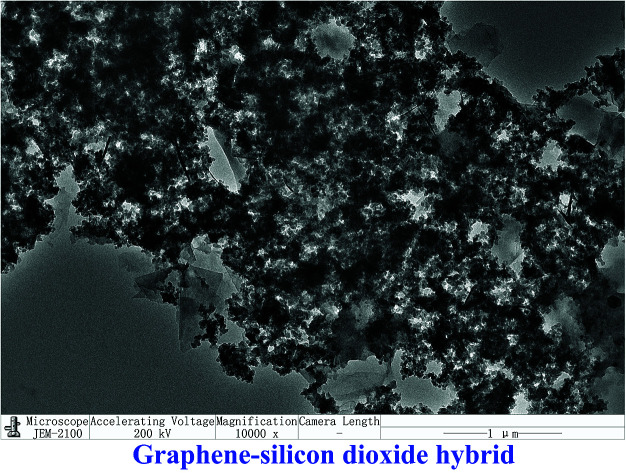

## 1. Introduction

High performance and superior interface interaction are always significant in polymer science and engineering, and stimulate numerous diverse applications of polymers. Generally, a single filler could not meet the variety of performance requirements of polymer products. Meanwhile, the fillers in a composite material system usually are poorly compatible with the polymer matrix. Recently, a hybrid filler consisting of two or more constituents with different performance and functions offers an alternative and novel inspiration for designing high-performance or functional polymer nanocomposites.^1–4^ Due to the specific interactions between different fillers, hybrid systems act with a positive synergistic effect on the overall performance of polymer nanocomposites.^2,5–7^

Nowadays graphene is widely used as functional filler in polymer nanocomposites for its extraordinary mechanical, thermal and electrical properties.^8–10^ Graphene can cause a crucial improvement in the electrical, mechanical, and thermal properties of the resulting graphene/polymer composites at very low doping percentages. However, graphene is an atomically thin nano-plate of sp^2^ bonded carbon atoms. It is a processing challenge for these hydrophobic fillers to be uniformly dispersed in the polymer matrix. Excellent dispersions and interfacial interactions between graphene and the polymers are vital to the generation and utilization of a strong composite.^11^ The prevalent strategy to intensify interactions between graphene and the polymer chains is chemical functionalization of the surface of the nanoparticles.^12^ However, the chemical functionalization strategies are extraordinary comprehensive and poor universal applicability, destruction of graphene intrinsic properties and that the interfaces interaction between graphene and the polymer chains is not obvious.

Recently, various hybrid combinations based on graphene have been supposed to be ideal candidates to construct hybrid fillers towards high-performance polymer nanocomposites. Metal oxides (TiO_2_, SnO_2_, Fe_3_O_4_)/graphene hybrids have been widely investigated as promising high-capacity anode material for lithium-ion batteries.^13,14^ A facile ionic self-assembly process has constructed two-dimensional graphene/SnO_2_/graphene hybrids with SnO_2_ nanoparticles sandwiched in graphene sheets.^15^ A nanocomposite comprised of chemically converted graphene and carbon nanotubes can preserve the intrinsic electronic and mechanical properties of both components.^16^ Baochun Guo *et al.* have systematically investigated the mechanism for the synergistic reinforcement in an elastomer reinforced by nanocarbon hybrids consisting of 2D reduced graphene oxide and 1D carbon nanotubes.^17^

While these achievements are quite impressive, synergistic effect of graphene and silicon dioxide (SiO_2_) hybrids in elastomer composites is still less research. SiO_2_ applied in composites have attracted much research attention^18–22^ in the last few decades owing to the superior properties of SiO_2_ nanoparticles such as toughness, tensile strength, thermal stability, gas separation performance, high scattered performance, and thermal resistance^23^ and they have various applications in high-performance coatings,^24,25^ fibers,^26,27^ bioactive polymers,^28,29^ rubber composites *etc.*^30–32^ The nanometer-sized SiO_2_ particles are used not only as a kind of filler, but also as reinforcing^33,34^ and toughening^35^ agents that can impart good mechanical properties, thermal stability, and toughness, as well as magnetic and optical properties to the polymers. However, it is usually hard to assure the uniform distribution of SiO_2_ because of its poor interaction with the organic matrix, which gives rise to poor stability of nanocomposites. Therefore, the strong interface adhesion between the organic matrix and SiO_2_ nanoparticles is a key to the application of SiO_2_ nanoparticles as fillers. Ming Tian *et al.* have prepared graphene oxide (GO)-encapsulated SiO_2_ hybrids *via* electrostatic self-assembly. The as-prepared hybrids were introduced into polydimethylsiloxane (PDMS) elastomer to simultaneously increase the dielectric constant (*k*) and mechanical properties of PDMS.^36^

SiO_2_/reduced graphene oxide hybrids were fabricated by an electrostatic assembly.^37^ The results revealed the better dispersion of hybrid filler and stronger interfacial interaction in the SBR and hybrid filler by contrast with those of SBR/SiO_2_ and SBR/reduced graphene oxide, contributes to the significant enhancement in the mechanical performances of composites. The achievement is quite significant. However, the hybrid filler system refers to SiO_2_ and reduced graphene oxide. Environmental pollution and performance damage of the preparation method for reduced graphene oxide limit its application greatly. Therefore, the research questions related to the synergistic effect of graphene and SiO_2_ hybrids in elastomer composites that need to be addressed.

**Scheme 1:**
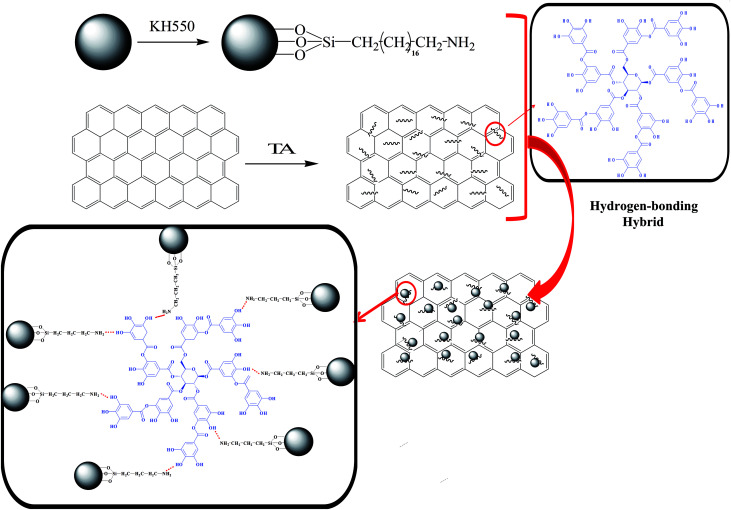
Schematic illustration of the synergistic hybridization graphene–SiO_2_ filler.

In this study, we aim to prepare a novel graphene–SiO_2_ hybrid (HGS) by a hydrogen-bonding assembly in favor of overcoming the aggregation of the individual graphene sheet and SiO_2_ nanoparticle; a schematic illustration is shown in Scheme 1. And subsequently, HGS was incorporated into NR to fabricate NR composites. It is effortless to guarantee not only the uniform distribution of HGS because of the synergistic disperse effect of graphene and SiO_2_ by a hydrogen-bonding assembly, but also the strongly interfacial interaction between HGS and polymer due to the increased surface roughness of graphene after loading SiO_2_. Furthermore, the intrinsic performances of graphene for G-TA are maintained well due to the physical interaction between graphene and TA. The overall performances of HGS@NR nanocomposites are improved drastically compared with that of nanocomposites prepared by dispersing the blend of GS in NR, proving the importance of the strong hydrogen-bonding interaction between phenolic hydroxyl groups on G-TA and primary amine groups on Si–NH_2_. As a result, the HGS@NR nanocomposites possess the highest tensile strength up to 27.8 MPa at 0.5 wt% and tear strength 60.2 MPa at 0.5 wt%. Thermal conductivities of the HGS@NR nanocomposites were found nearly 1.5-fold better than that of the NR/HGS nanocomposites. The HGS@NR nanocomposites exhibit excellent abrasive resistant capacity with nearly 2-fold better than that of the GS@NR nanocomposites.

## 2. Experimental

### 2.1 Materials

Graphene was supplied by the sixth element (Changzhou) material technology Co. Ltd. (China). Submicron-sized SiO_2_ (average diameter: 300 nm) was supplied by Degussa AG (Germany). γ-Aminopropyltriethoxysilane (KH550) was purchased from Sigma-Aldrich Technology Co., LTD (China). Plant polyphenol-tannic acid (TA) was purchased from Aladdin Technology Co., LTD (China). NR was provided by Sanlux Co., Ltd (China). Zinc oxide, stearic acid, *N*-isopropyl-*N*′-phenyl-*p*-phenylenediamine (4010NA), 2-benzothiazolyl disulfide (DM) and *N*-cyclohexyl-2-benzothiazole sulfenamide (CZ) were provided by Sanlux Co., Ltd (China).

### 2.2 Hydrophobic modification of SiO_2_ particles

The SiO_2_ particles were modified with silane coupling agent γ-aminopropyltriethoxysilane, KH550.^38^ The mass ratio of coupling agents relative to SiO_2_ is 0 : 20, 1 : 20, 2 : 20 and 3 : 20. Under vigorous stirring (5000 rpm), a dosage of coupling agents (0 g, 1 g, 2 g and 3 g) was respectively dispersed with a mixture solvent (1 mL deionized water and 10 mL ethanol) and added into four different three-necked round-bottom flasks. The reaction was carried out at 50 °C for 30 min for the completion of the hydrolysis reaction. Then a fixed mass SiO_2_ (20 g) was respectively added into the four three-necked round-bottom flasks mentioned above and vigorously stirred at 5000 rpm at 60 °C for 3 h. The silane coupling agents modified SiO_2_ nanoparticles were washed with ethanol for three times after the centrifugation separation. The white powder was dried in a vacuum oven at 40 °C for 12 h and designed as 0S, 1S, 2S, and 3S.

### 2.3 Preparation of graphene–SiO_2_ hybrid

3 g of graphene was added into a vial containing 30 mL TA aqueous solution which the concentration of TA is 0.05 g mL^−1^. The mixture was then sonicated for 30 min in a 30–35 °C water bath using a bath sonicator (Elmasonic E30H, 40 W, 37 kHz) to prepare TA functionalized graphene. Then TA functionalized graphene disperse is equally divided into triplicate. Subsequently, all the above prepared 1S, 2S and 3S was respectively added to the three TA functionalized graphene aqueous solution with the same volume, and then the mixtures were sonicated in a 30–35 °C water bath for 1 h and designed as H1GS, H2GS, H3GS respectively. For comparison purpose, a sample of unmodified graphene–SiO_2_ hybrid (designed as GS) was prepared from 0S and unmodified graphene subjecting to the same procedures that were used to prepare H1GS.

### 2.4 Preparation of rubber/graphene–SiO_2_ hybrid composites

Composite was performed in a 200 mL Banbury mixer at a rotor speed of 60 rpm for the mixing stage at a temperature of 120 °C. The NR was fed into the mixer and premixed for 2 min. This was followed by the sequential addition of zinc oxide (5 phr), stearic acid (3 phr), 4010NA (3 phr), DM (0.1 phr) and CZ (1.4 phr) and mixing the compounding ingredients for another 4 min. Next our above prepared graphene–SiO_2_ hybrid fillers (H1GS) was all added and compounded into the rubber for 3 min and then the mixture was discharged onto a two roll mill at 80 °C and designed as H1GS@NR, where the sulfur (2.8 phr) was added. H2GS@NR and H3GS@NR were prepared from H2GS, H3GS and NR subjecting to the same procedures that were used to prepare H1GS@NR. For comparison purpose, a sample of NR/unmodified graphene–SiO_2_ hybrid composite (designed as GS@NR) was prepared from GS and NR subjecting to the same procedures that were used to prepare H1GS@NR.

### 2.5 Characterization

The infrared spectra were obtained with a Bruker Vertex 70 variable temperature Fourier Transform Infrared Spectrometer. Zeta potential (*ζ*) was measured on a Malvern Zetasizer Nano-ZS system equipped with a 632.8 nm He-Ne laser. Thermogravimetric analysis was performed with a TGA 209 F1 instrument (NETZSCH) with a heating rate of 10 °C min^−1^ under nitrogen purging. X-ray diffraction (XRD) pattern was recorded on a Rigaku D-MAX2500-PC diffractometer. Transmission electron microscopy (TEM) images were obtained using a JEM-2100 microscope. Scanning electron microscopy (SEM) was performed with JEOL-JSM-7500F microscopy. Dynamic mechanical analysis was performed on a DMA242 machine (NETZSCH) in tensile mode with a temperature increment of 3 °C min^−1^. Dynamic rheological tests were used to measure the dynamic viscoelastic properties of hybrid nanocomposites by Rubber Processing Analyzer (RPA 2000, Alpha Technologies) with strain sweeps performed from 0.25% to 125% at a frequency of 1 Hz and at a temperature of 333 K. The thermal conductivity was measured by a laser flash system (LFA 447 NanoFlash). Tensile and tear testing were carried out using an AI-7000S Universal Material Tester, with a dumbbell specimen at a tensile speed of 500 mm min^−1^ according to ISO 528:2009. Compression temperature rise and compression set are determined at 55 °C using rubber compression heat tester. The abrasion test was carried out with the Akron abrader according to GT-7012-A. The abrasion was defined as a volumetric loss of a strip sample.

## 3. Results and discussion

### 3.1 The chemical structure of Si–NH_2_ and G-TA

**Fig. 1:**
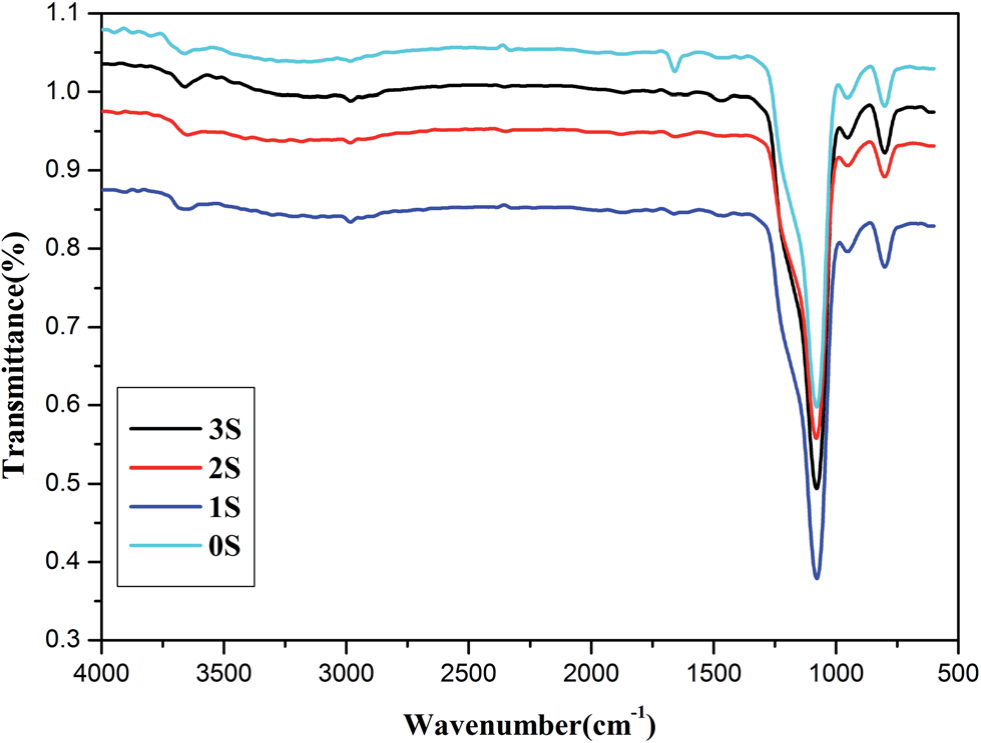
FTIR spectrum of 0S, 1S, 2S and 3S.

The FTIR spectra of 0S, 1S, 2S and 3S, are shown in Fig. 1. The peak at 1107 cm^−1^ [Fig. 1 (0S)] corresponds to the asymmetric stretching vibration of Si–O–Si, and the peak at 3423 cm^−1^ is the characteristic absorption peak of –OH groups. Compared with 0S, the FTIR spectrum in [Fig. 1 (1S, 2S, 3S)] shows that the peak at 2929 cm^−1^ corresponds to the asymmetric stretching vibration of –CH_2_, and the peaks at 1453 cm^−1^ correspond to the asymmetric bending vibration of –CH_2_, respectively, indicating that KH550 is successfully grafted onto SiO_2_. Moreover, the strong peak at 1640 cm^−1^ (0S) which is attributed to the bending vibration of H_2_O is represented weaker and weaker for 1S, 2S and 3S. It indicated that the hydrophobicity of SiO_2_ after modified with KH550 becomes strong. Just the FTIR spectra of 3S show that the double peaks between 3300 cm^−1^ to 3500 cm^−1^ which represent the characteristic absorption peaks for the stretching vibration of the primary amine group. It can be expected that the strong signal peaks of SiO_2_ mask the much weaker signal peaks of primary amine group due to low grafting degree.

**Fig. 2:**
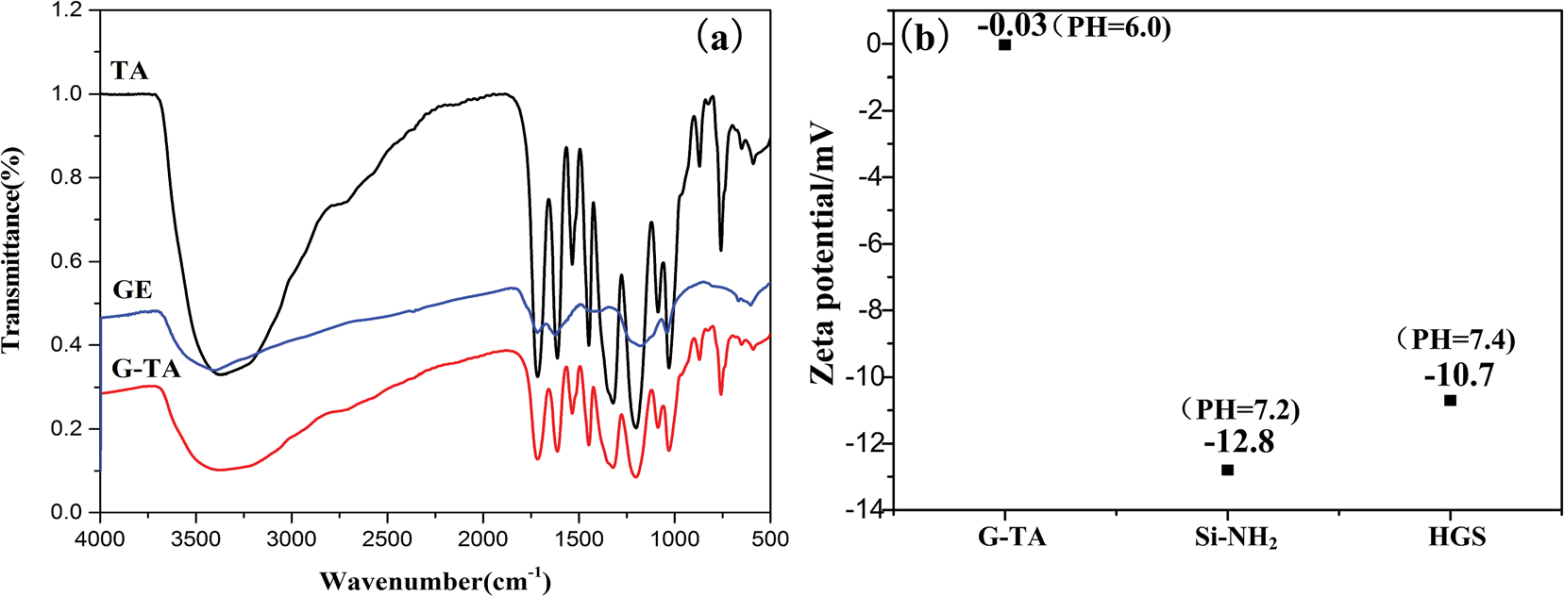
(a) FTIR spectra of graphene (GE), TA and G-TA; (b) zeta potentials of G-TA, Si–NH_2_ and H3GS.

**Fig. 3:**
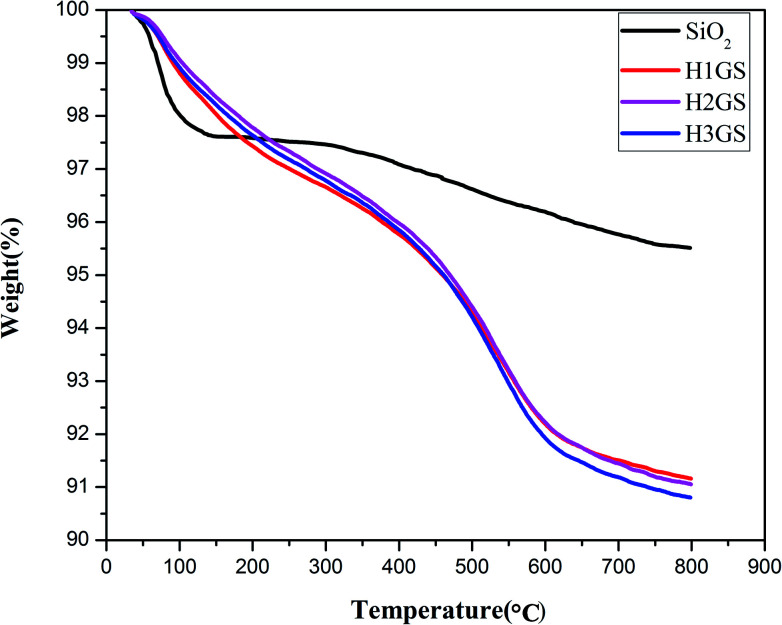
TGA graph of H1GS, H2GS and H3GS.

To make clear the interaction between TA and graphene, FTIR has been performed to characterize the obtained G-TA hybrid. As illustrated in the FTIR spectra in Fig. 2a, compared with the spectrum of TA, G-TA has a similar spectrum with TA, no new signal peaks appear, but the characteristic peaks of TA such as O–H bending vibration (1310 cm^−1^), aromatic ring breathing vibration (1640 and 1525 cm^−1^), are significantly blue shifted to 1383, 1670 and 1570 cm^−1^, respectively. The broad peak at 3310 cm^−1^ [Fig. 2a (G-TA)] in the spectra is associated with OH groups attached to TA, which demonstrating the absorbance of TA on graphene. Therefore, the characteristic peaks of G-TA come from TA noncovalently absorbed on graphene *via* strong physical interaction, which is acknowledged to be the π–π interaction between plenty of aromatic rings of TA and graphene.^39–41^ The zeta potentials of the G-TA, Si–NH_2_ and H3GS dispersions respectively reach −0.03 mV, −12.8 mV and −10.7 mV (Fig. 2b), indicating the hybrid effect of graphene and SiO_2_ stems from the hydrogen bonding supplied by TA and primary amine rather than the electrostatic repulsion. Meanwhile, TGA analysis was used to quantitatively evaluate the hybrid fillers, as illustrated in Fig. 3. The hybrid fillers start to decomposition before 300 °C which is relative to the degradation of tannic acid and water. The weight loss in the temperature range of 300–600 °C manifests the condensation of the silanol groups on the surface of SiO_2_. The residual weight implies that the grafted KH550 content onto the SiO_2_ for H1GS, H2GS and H3GS are about 4.35 wt%, 4.46% and 4.71% respectively.

### 3.2 Microstructure of hybrid filler system

**Fig. 4:**
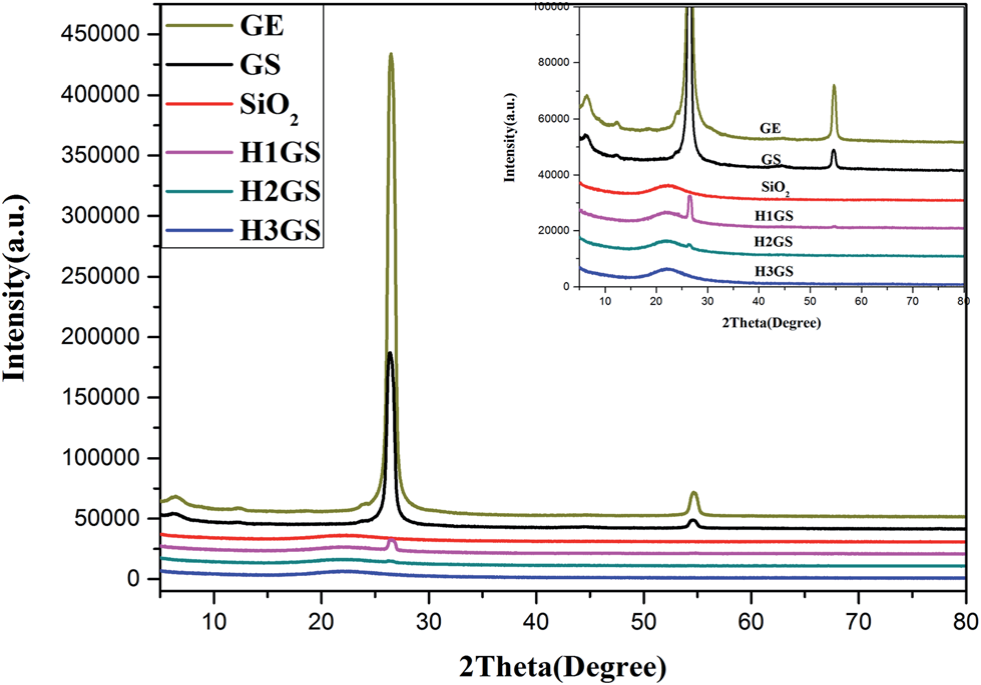
XRD spectra of graphene (GE), SiO_2_, GS, H1GS, H2GS and H3GS.

The XRD patterns of graphene, SiO_2_, GS and H1GS, H2GS, H3GS were shown in Fig. 4. The major peaks at 2*θ* = 26.2° and 54.8° are due to (002) and (004) of graphite and indicate the multilayer structure in graphene. The intensities of the major peak (002) and (004) monitor the disordered structure and defects inside graphene and hence provides implication for the dispersion status of hybrid filler. And that notes that the broadness of the major peak suggests the presence of small irregularity in the interplanar distances in (002).^42^ The GS sample demonstrates similar XRD diffraction peaks of graphene, even though the intensities of the major peak (002) and (004) are much weaker, and the broadness of (002) is much wider than that of graphene, which displaying the still existence of multilayer aggregation structure of graphene and poor dispersion even after van der Waals force hybridization process. The XRD diffraction peaks (002) and (004) corresponding to graphene cannot be seen in H1GS, H2GS, and H3GS, indicating that the disaggregation of graphene and the thickness of graphene is less than 3 nm.^43^ Moreover, it indicates higher hybridization on the surface of hybrid fillers are detected, most likely due to hydrogen bonding interaction between T-GA and Si–NH_2_. Furthermore, the vanishment of the peaks (002) and (004) of H1GS, H2GS, H3GS is also indicative of a strong interfacial interaction between graphene and SiO_2_. In our study, this is probably because the Si–NH_2_ nanoparticles hinder the graphene nanosheets from restacking, disrupt the ordered lamellar structure, and accordingly result in the vanishment of the diffraction peaks of graphene through hydrogen bonding self-assembly hybrid effect.

**Fig. 5:**
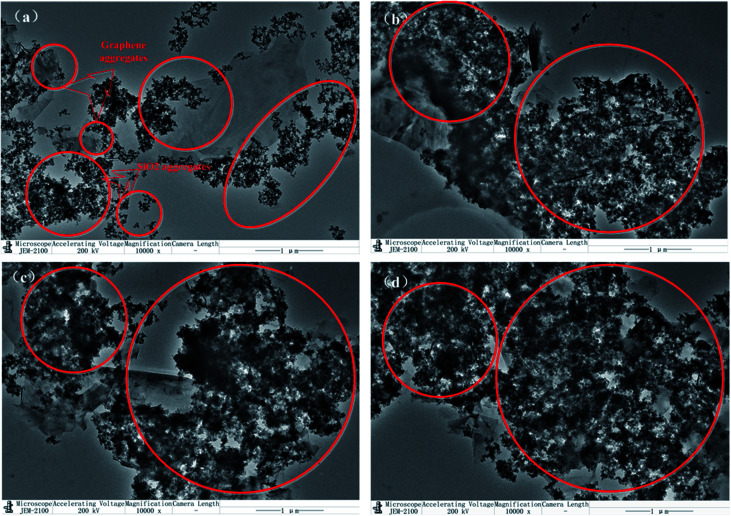
TEM images of (a) GS, (b) H1GS, (c) H2GS and (d) H3GS.

To further demonstrate the microscopic distribution state of hybrid fillers, TEM images as depicted in Fig. 5. H1GS, H2GS, and H3GS (Fig. 5b–d) demonstrate that the SiO_2_ nanoparticles are uniformly decorated on the surface of the graphene and well distributed throughout the surface of graphene for them. Moreover, the surface of H1GS, H2GS and H3GS is much rougher than that of GS (Fig. 5a). And that there is the more uniform distribution density of SiO_2_ and much higher surface roughness of hybrid fillers with higher silane coupling agent levels. Compared with GS, H1GS, H2GS and H3GS revealed the covering of entire graphene sheet by coating due to hydrogen bonding interaction. However, along with the wrinkles and edges of graphene, the SiO_2_ nanoparticles of GS (Fig. 5a) are only attached to the graphene nanosheets. It is because that unmodified SiO_2_ is easier to form weak van der Waals force hybridization interactions with unmodified graphene along the wrinkles and edges of graphene. After earliest attaching of a small amount of SiO_2_ nanoparticles in these areas, the rest of nanoparticles tend to form agglomeration within these regions. Thus, in GS sample with weak van der Waals force, graphene is barely covered with SiO_2_ particles (Fig. 5a) along with phase separation from SiO_2_. It is important to note that the hydrogen-bonding between TA and silane coupling agent plays a role of surfactant to determine the interfacial interactions between graphene and SiO_2_ in promoting the formation of graphene–SiO_2_ hybrid nanostructures. TEM micrographs confirm that HGS is few-layer graphene depicted in XRD results.

### 3.3 Dispersion state of hybrid filler system in NR composites

**Fig. 6:**
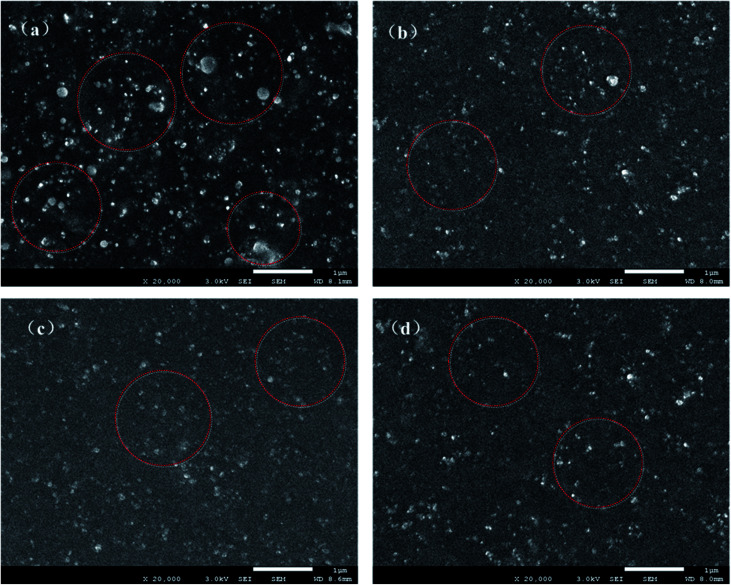
SEM images of (a) GS@NR, (b) H1GS@NR, (c) H2GS@NR and (d) H3GS@NR composites.

To characterize the microdispersion state of the fillers upon hybridization, SEM images of GS@NR and HGS@NR composites with different KH550/TA ratios are shown in Fig. 6. Even after sonication for a long time, a van der Waals force hybrid filled system (GS) cannot form a uniform distribution in NR matrix in which large aggregates with several microns (as indicated by the red circle) in diameter and are exposed all around the fracture surface. This clearly demonstrates the poor filler dispersion and filler–polymer interaction in GS@NR composites. As for hydrogen bonding self-assembly hybrid fillers (HGS), the filler dispersion state and compatible state are significantly improved as shown in Fig. 6b–d. Evidently, HGS with a size of about hundreds of nanometer (as indicated by the red circles) disperses homogeneously in NR matrix. The dispersal state of HGS was improved with increasing the KH550/TA ratio.

Moreover, from Fig. 6b–d, we can see clearly that the HGS@NR nanocomposites display smooth surface morphology. When the KH550/TA ratio increases, the roughness degree of HGS@NR nanocomposite becomes smaller, which contribute to the stable dispersion of HGS nanoparticles in NR matrix and good compatible of HGS nanoparticles with NR matrix due to the low surface energy of HGS through hydrogen bonding self-assembly hybridization. Hence, it's reasonable to believe that the significantly improved hybrid filler dispersion in NR matrix originated from the strong hydrogen bonding self-assembly interaction between graphene and SiO_2_. The synergistic intercalation between the graphene nanoplatelets and the spherical SiO_2_ can inhibit their re-agglomeration and form strong network architecture.

### 3.4 Effect of hybrid filler network on the chain relaxation dynamics of NR composites

**Fig. 7:**
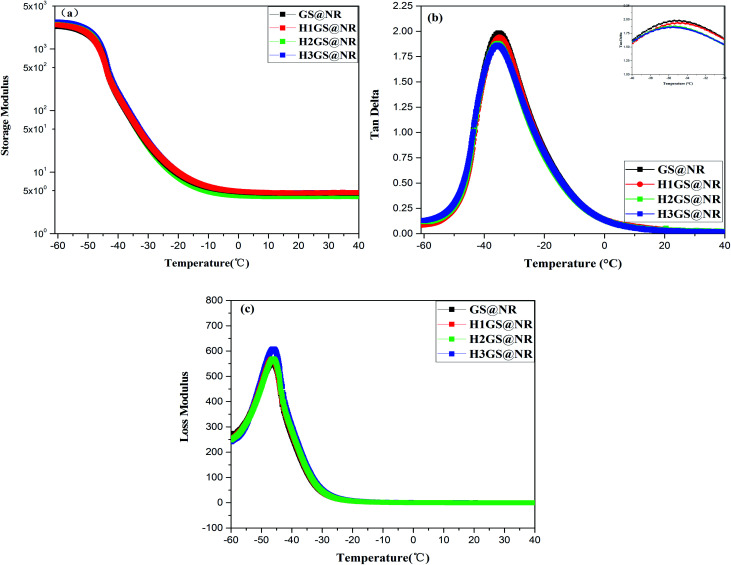
(a) Storage modulus (*G*′), (b) loss factor (tan *δ*) and (c) loss modulus (*G*′′) *versus* temperature for GS@NR and HGS@NR composites.

The synergistic intercalation due to the formation of strong network architecture is demonstrated by dynamical mechanical analysis. The results of the measurements of storage modulus (*G*′), and loss factor (tan *δ*) are given in Fig. 7. When nanoparticles are added to the polymer, as the content of nanoparticles increases, the material will gradually change from similar liquid state to similar solid state. In dynamic viscoelastic research, loss factor (tan *δ*) is usually used to characterize the interaction between filler and polymer as well as filler and filler. The viscoelasticity of hybrid nanocomposites was studied and the response signal of different loss factors was found in GS@NR and HGS@NR nanocomposites.

The tan *δ* (Fig. 7a) of HGS@NR composites is much lower than that of GS@NR. Moreover, the lower the tan *δ* of HGS@NR composite is, the higher the content of the KH550/TA ratio is synergistic intercalation between the SiO_2_ and graphene can inhibit their re-agglomeration and form tight network architecture. Previous studies have shown that the incorporation of all kinds of nanocarbon gives rise to a decline in the peak value of tan *δ*.^44^ Hence, it's reasonable to believe that the significantly improved hybrid filler dispersion in NR matrix originated from the strong hydrogen bonding self-assembly interaction between Si–NH_2_ and G-TA. Moreover, it's reasonable to suppose that a more efficient formation of hybrid filler network can generate an exclusive relaxation dynamics of polymer chains. The confinement effect on the NR matrix by graphene–SiO_2_ hybrid filler network can also be evaluated by storage modulus (Fig. 7b). It should be noted that, the larger increases in *G*′ are attained in the HGS@NR over the entire temperature range, as compared with GS@NR. This finding can be explained by fact that the enhanced constrained region in the HGS@NR can be in favor of a very efficient load transfer from the matrix to HGS hybrid filler. That is, the increased fraction of the constrained region can markedly contribute to improving the performance of NR composites.

**Fig. 8:**
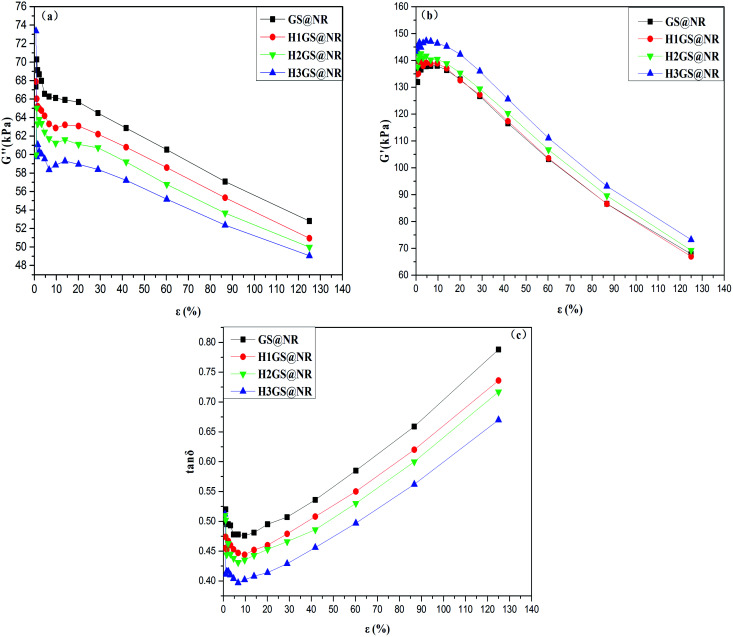
(a) Strain-dependent loss modulus, (b) storage modulus and (c) tan *δ* curves of GS@NR, H1GS@NR, H2GS@NR and H3GS@NR composites.

Rubber Processing Analyzer is also employed to evaluate the effect of the filler network on the thermal relaxation of NR chains as shown in Fig. 8. Fig. 8c depicts the strain-dependent tan *δ* curves of NR composites with different fillers. The peak value of tan *δ* tends to decrease with increasing HGS filler content. Moreover, the values for the HGS@NR composites are much higher than those for the GS@NR at the same strain. Such results suggest that the enhanced interfacial interaction between HGS and the matrix can effectively prompt the configurational constraints of the NR chains, improving the fraction of constrained region in the rubber composites. It's widely accepted that the peak value of tan *δ* is a measurement of the energy dissipation in polymer composites during the mechanical-cyclic test.^45,46^ Such energy dissipation is inversely proportional to the interfacial interaction between polymer and filler network which will substantially constrain and impair the mobility of polymer chains.^47^

The results of the measurements of loss modulus (*G*′′) (Fig. 8a) show that the linear viscoelastic region decreases slightly with the interfacial interaction between the polymer and the filler network becomes strong. Meanwhile, the presence of a rigid three-dimensional filler network in the rubber composites can be characterized by the non-linearity of the viscoelastic storage modulus (Fig. 8b) at small dynamic strain amplitudes, knowing as the Payne effect.^48^ The Payne effect declined as the HGS hybrid filler content increased, indicating a reduction in filler network intensity, which demonstrating a superior reinforcement capability of HGS hybrid filler.^37^

### 3.5 Thermal and mechanical properties of NR composites

**Fig. 9:**
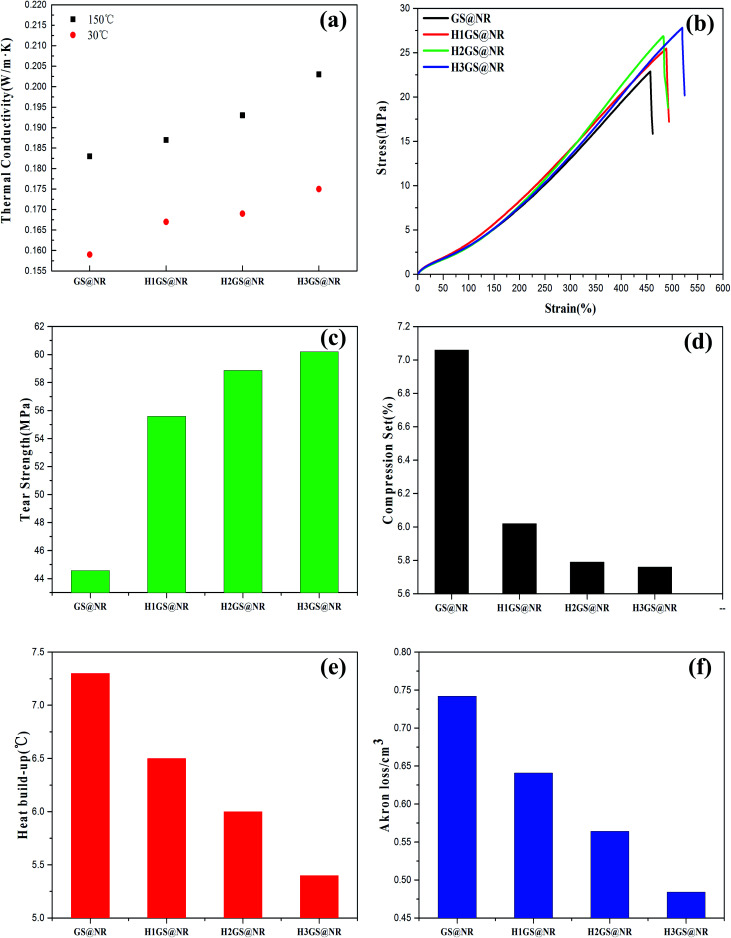
(a) Thermal conductivity of GS@NR and HGS@NR composites, (b) tensile strength of GS@NR and HGS@NR composites, (c) tear strength of GS@NR and HGS@NR composites, (d) compression set of GS@NR and HGS@NR composites, (e) compression temperature rise of GS@NR and HGS@NR composites, (f) abrasive resistance of GS@NR and HGS@NR composites.

Concerning with thermal conductivity, compared with GS@NR, this was found to be affected by hybridization forms, where hydrogen bonding self-assembly hybrid interaction resulted in a significant increase in thermal conductivity of composites attributes from the nearly complete connected graphene sheets (Fig. 9a). On the one hand, a certain part of evenly distributed SiO_2_ is used to bridge the graphene sheet gaps in the in-plane direction that enlarge the phonon transmission channel and benefit the phonon propagation, while the re-aggregation of SiO_2_ induces larger interfacial thermal resistance that further decreases the thermal conductivity of GS@NR.^49^ On the other hand, previous studies^50–52^ indicated that when graphene sheets are over stacked into a paper, its thermal conductivity greatly drops one order of magnitude due to the phonons leakage across the interface and the enhanced umklapp scattering of out-plane acoustic phonons. However, graphene layers in HGS hybrid fillers are intercalated by SiO_2_ particles. Graphene sheets are partly separated by SiO_2_ that effectively retards the strong graphene interlayer stacking induced by the strong π–π interactions. Thus phonons transfer thermal energy in the strictly 2D channel with the interference from out-of-plane being eliminated. It also means that effective thermal conduction path of graphene can be formed. The performance of HGS@NR composites would, therefore, be superior to that of GS@NR. Furthermore, thermal conduction path of graphene is more accessible to create promptly with increasing the ratio of KH550 to TA.

The results obtained for the mechanical properties of the variously compounded samples are presented in Fig. 9b–f. The HGS@NR composites deal with effects of change in the ratio of KH550 to TA at a fixed ratio of SiO_2_ to graphene. It is clearly seen from the Fig. 9b that the tensile strength range is large varying between a minimum of 22.73 MPa to a maximum of 27.59 MPa. It is also seen from Fig. 9c that the highest tear strength of 60.20 MPa is with H3GS@NR followed by H2GS@NR showing a value of 58.87 MPa. However, with the compound containing GS, the tear strength is just the value at 44.57 MPa. As for HSG@NR composites, namely H1GS, H2GS and H3GS, the compression set, compression heat, flexing resistance and abrasive resistance properties not only improve but vary within wide limits. So compared with GS@NR composite, the comprehensive mechanical properties for HGS@NR composites to judge interface interaction fitness of composites are all promoted. The reason can be explained through their physical structures, size, shape, surface area and anisotropy of their distribution of hybrid fillers in the polymer matrix.^53^ The formation of tight network architecture due to synergistic hydrogen bonding self-assembly can prompt hybrid fillers to disperse well in the polymer matrix and prevent the stress concentration effectively, improving physical properties comprehensively. Furthermore, the formation of a glassy interphase with orders of magnitude slower chain dynamics than that for bulk chains has been explicitly demonstrated in the synergistic hydrogen bonding self-assembly hybrid system.^17^ Therefore, besides improved dispersion upon hybridization, it is believed the formation of a glassy interphase is another crucial factor in governing the synergistic capability of hybrid composites, as the showed results in DMA (Fig. 7) and RPA (Fig. 8). It can be explained also that graphene–SiO_2_ hybrid filler by hydrogen bonding self-assembly interaction had higher surface roughness values than that after van der Waals force hybridization.^54^ Meanwhile, the surface roughness is much higher with increasing the ratio of KH550 to TA as shown in Fig. 4. The surface roughness of fillers plays an important role in governing interfacial interaction between fillers and polymer matrix. This can be explained by the significantly higher maximum contact pressures for rough surfaces, which are present at the surface asperities.^55^ During compounding, due to high contact pressure, the sharp and hard protruding surface asperities of the rough hybrid fillers cause considerable interfacial interaction, leading to unique dispersion in polymer matrix. The strain–stress curve also indicates the discrepancy in toughness among the four nanocomposites. Here toughness is defined by the area of strain–stress curve which represents the absorbing energy without fracturing. H1GS@NR, H2GS@NR and H3GS@NR lead to improved toughness compared to GS@NR. Moreover, with increasing the ratio of KH550 to TA, it exhibits a significant increase in toughness. This is attributed to the improved dispersion and strong interface interaction between HGS and NR upon hydrogen bonding self-assembly hybridization. This leads to higher energy absorption and higher toughness.

## 4. Conclusion

In conclusion, a novel method for fabricating graphene–SiO_2_ hybrid filler through hydrogen bonding self-assembly interaction has been demonstrated. The SiO_2_ nanoparticles are uniformly decorated on the surface of the graphene and are well distributed throughout the surface of graphene. The formation of tight network architecture due to synergistic hydrogen bonding self-assembly intercalation effect between silane coupling agents modified SiO_2_ and TA modified graphene can prompt hybrid fillers to disperse well in the polymer matrix and prevent the stress concentration effectively, improving comprehensive performances. An effective thermal conduction path of graphene for HGS@NR can be formed to dramatically improve thermal property of composites. The superior synergistic hydrogen-bonding interaction can make the filler–rubber interfacial interaction strongly, which, in turn, contributes to the significant enhancement in the mechanical performances of composites by the incorporation of graphene–SiO_2_ hybrid filler into NR matrix. Also, it was noted that HGS has a higher reinforcing effect than those of GS. Particularly, the tensile strength and tear strength for H3GS@NR are dramatically increased by about 21.4% and 35.0% as compared with those of GS@NR, respectively.

## Conflicts of interest

There are no conflicts to declare.

## Supplementary Material

RA-008-C8RA01659C-s001
