# Supplementary material for: Synergistic effect of graphene and silicon dioxide hybrids through hydrogen bonding self-assembly in elastomer composites
Source: RSC Adv. 2018 May 16;8(32):17813–25. doi: 10.1039/c8ra01659c (PMC9080488; doi:10.1039/c8ra01659c)
Supplement: RA-008-C8RA01659C-s001 [file RA-008-C8RA01659C-s001.pdf]

Electronic Supplementary Material (ESI) for RSC Advances.
This journal is © The Royal Society of Chemistry 2018

Supporting information

Synergistic effect of graphene and silicon dioxide hybrids through hydrogen bonding self-assembly in elastomer composites

Shuai Zhao*, Shicheng Xie, Peipei Sun, Zheng Zhao, Lin Li*, Xiaoming Shao, Xiaolin Liu, Zhenxiang Xin

Table S1 Thermal conductivity raw data for GS@NR and HGS@NR nanocomposites

Shot number	Temperature（℃）	Thermal Conducitvity(W/m*K)
GS@NR	30.4	0.160
	30.0	0.165
	29.8	0.158
	30.0	0.156
	30.1	0.157
Mean	30.1	0.159
H1GS@NR	29.7	0.162
	30.0	0.165
	30.1	0.172
	30.0	0.177
	30.0	0.160
Mean	30.0	0.167
H2GS@NR	29.9	0.165
	30.1	0.187
	30.0	0.158
	30.1	0.170
	30.1	0.167
Mean	30.0	0.169
H3GS@NR	29.7	0.204
	30.1	0.160
	30.1	0.184
	30.0	0.164
	30.0	0.161
Mean	30.0	0.175
GS@NR	149.8	0.185
	149.9	0.187
	150.1	0.186

	150.0	0.180
	150.1	0.177
Mean	150.0	0.183
H1GS@NR	149.9	0.164
	149.7	0.200
	150.0	0.197
	150.1	0.189
	150.1	0.185
Mean	150.0	0.187
H2GS@NR	150.0	0.195
	150.1	0.204
	150.0	0.185
	150.1	0.191
	150.1	0.190
Mean	150.0	0.193
H3GS@NR	150.1	0.231
	150.0	0.196
	150.0	0.193
	150.0	0.210
	150.0	0.187
Mean	150.0	0.203
